# The role of the blood–brain barrier in the development and treatment of migraine and other pain disorders

**DOI:** 10.3389/fncel.2014.00302

**Published:** 2014-10-08

**Authors:** Marcos F. DosSantos, Rosenilde C. Holanda-Afonso, Rodrigo L. Lima, Alexandre F. DaSilva, Vivaldo Moura-Neto

**Affiliations:** ^1^Universidade Federal do Rio de Janeiro – Campus MacaéRio de Janeiro, Brazil; ^2^Laboratório de Morfogênese Celular, Instituto de Ciências Biomédicas, Universidade Federal do Rio de JaneiroRio de Janeiro, Brazil; ^3^Headache and Orofacial Pain Effort, Department of Biologic and Materials Sciences and Michigan Center for Oral Health Research, School of Dentistry, University of MichiganAnn Arbor, MI, USA; ^4^Departamento de Ortodontia e Odontopediatria, Faculdade de Odontologia, Universidade Federal do Rio de Janeiro, Rio de JaneiroBrazil; ^5^Instituto Estadual do Cérebro Paulo NiemeyerRio de Janeiro, Brazil

**Keywords:** pain, blood–brain barrier, blood–nerve barrier, blood–spinal cord barrier, neuropathic pain, migraine, inflammatory pain and opioids

## Abstract

The function of the blood–brain barrier (BBB) related to chronic pain has been explored for its classical role in regulating the transcellular and paracellular transport, thus controlling the flow of drugs that act at the central nervous system, such as opioid analgesics (e.g., morphine) and non-steroidal anti-inflammatory drugs. Nonetheless, recent studies have raised the possibility that changes in the BBB permeability might be associated with chronic pain. For instance, changes in the relative amounts of occludin isoforms, resulting in significant increases in the BBB permeability, have been demonstrated after inflammatory hyperalgesia. Furthermore, inflammatory pain produces structural changes in the P-glycoprotein, the major eﬄux transporter at the BBB. One possible explanation for these findings is the action of substances typically released at the site of peripheral injuries that could lead to changes in the brain endothelial permeability, including substance P, calcitonin gene-related peptide, and interleukin-1 beta. Interestingly, inflammatory pain also results in microglial activation, which potentiates the BBB damage. In fact, astrocytes and microglia play a critical role in maintaining the BBB integrity and the activation of those cells is considered a key mechanism underlying chronic pain. Despite the recent advances in the understanding of BBB function in pain development as well as its interference in the efficacy of analgesic drugs, there remain unknowns regarding the molecular mechanisms involved in this process. In this review, we explore the connection between the BBB as well as the blood–spinal cord barrier and blood–nerve barrier, and pain, focusing on cellular and molecular mechanisms of BBB permeabilization induced by inflammatory or neuropathic pain and migraine.

## INTRODUCTION

**FIGURE 1:**
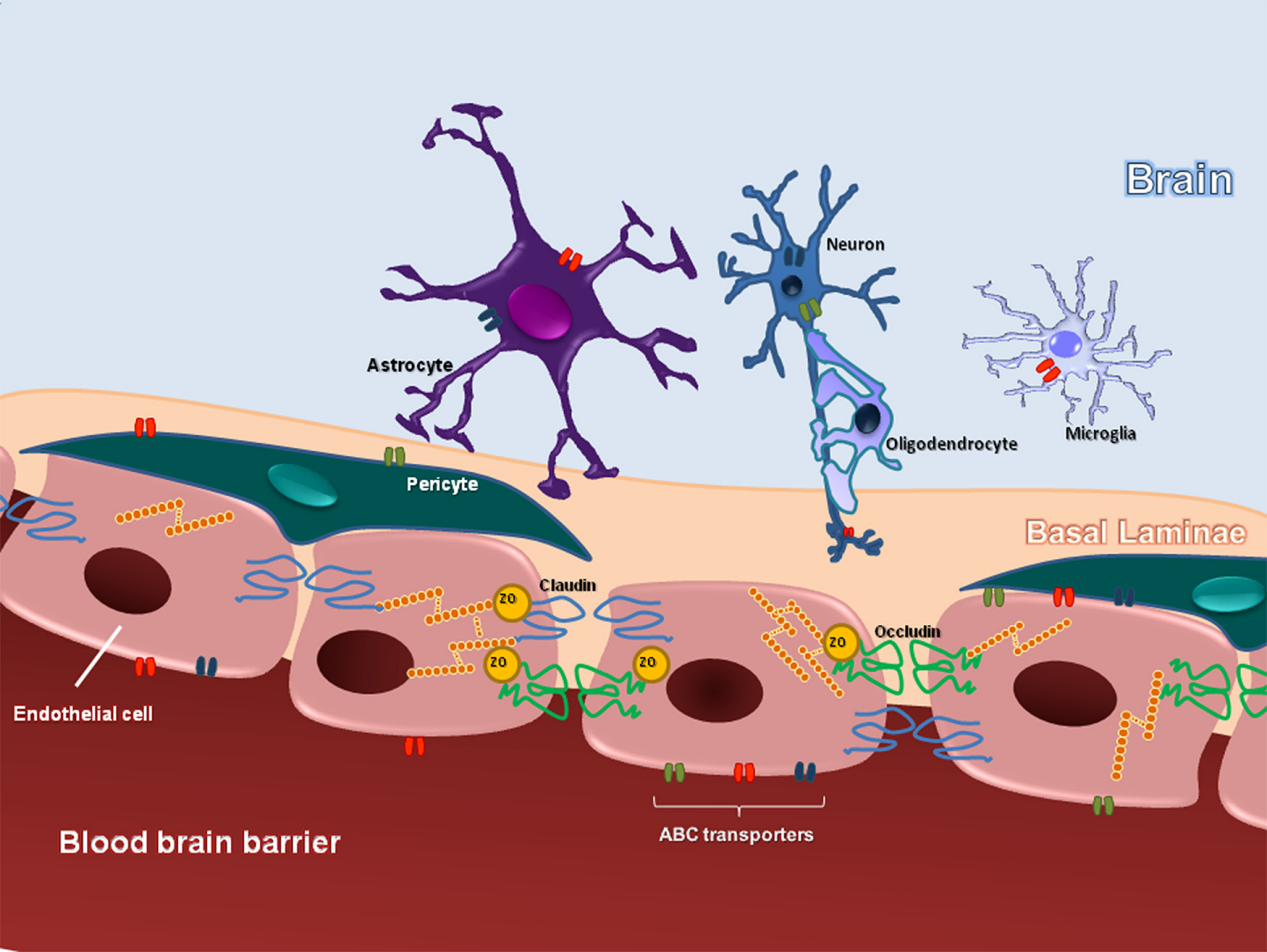
**Schematic representation, illustrating the basic structural organization of BBB**.

The BBB is referred as a dynamic and functional structure that separates the systemic circulation from the CNS. The BBB has a crucial role in maintaining the proper neuronal function. It is responsible for the brain homeostasis and protects the nervous tissue from potential harmful substances, by limiting the entry of certain molecules (except the small and lipophilic) into the CNS (Rubin and Staddon, 1999). The “neurovascular unit” comprises the endothelial cells, pericytes, and astrocytes endfeet, embedded within their basal laminae. The interface between blood and CNS is represented by the space between endothelial cells/pericytes and astrocytic endfeet (Beggs et al., 2010). BBB acts as a selective barrier due to the presence of complex TJs, located between adjacent endothelial cells (Abbott et al., 2006). The TJ protein complex establishes a physical barrier and limits paracellular diffusion (Sanchez-Covarrubias et al., 2014). It is formed via an intricate communication of transmembrane, accessory, and cytoskeleton proteins. The transmembrane proteins occludin and claudins are considered the primary seal of the TJ (Fricker and Miller, 2004; Hawkins and Davis, 2005) and dynamic interactions with the accessory proteins ZO 1, 2, 3 permit the connection between TJ and the actin cytoskeleton (Tsukamoto and Nigam, 1997). The biochemical barrier in the BBB comprises mainly influx and eﬄux transporters, located in the luminal and abluminal membranes of capillary endothelial cells as well as metabolizing enzymes expressed intracellularly (Hawkins and Davis, 2005; Ronaldson and Davis, 2013). ABC transporters are among the largest family of transmembrane proteins. They include P-glyprotein (P-gp), BCRP in humans and Bcrp in rodents, and MRP 1–6 in humans and Mrp 1–6 in rodents (Ronaldson and Davis, 2011; Radu et al., 2013). The main structures that compose the BBB are illustrated in the **Figure 1**.

Not all areas in the brain contain a BBB. Some areas where the BBB is absent are: hypophysis, median eminence, area postrema, preoptic recess, paraphysis, pineal gland, and endothelium of the choroid plexus (Siegel, 1999). In the spinal cord, the interface between blood and neural tissue is formed by the BSCB functionally equivalent to the BBB (Xanthos et al., 2012), while in the peripheral nerve, the perineurium, and the endothelial blood vessels form the BNB. The BNB also acts as a semipermeable membrane, regulating the microenvironment homeostasis and providing “privileged” space for peripheral axons and the corresponding supporting cells (Kanda, 2013; Lim et al., 2014).

It has been reported that the BBB morphology and function might be modulated and even disrupted in many neurological diseases, including those caused by extrinsic factors, such as meningitis (bacterial and viral) and encephalitis (e.g., herpes virus); intrinsic factors, such as ischemia/hypoxia, traumatic brain injury, small vessel diseases (e.g., hypertension, diabetes), and Alzheimer’s Disease; and more recently by pain disorders, including peripheral inflammatory pain, neuropathic pain, and migraine (Rosenberg, 2012). Tissue damage can produce an intense release of signaling molecules from peripheral and central neurons as well as from blood cells. Those substances include IL-1β, TNF-α, histamine, and fractalkine. Moreover, other substances are released at the site of the injury, such as serotonin, substance P, CGRP, and ATP. These are neurotransmitters of primary sensory afferents and are not only released during tissue injury (Abbott et al., 2006; Basbaum et al., 2009; Clark and Malcangio, 2014). Many such mediators can generate significant effects in the CNS barriers (BBB, BSCB, and/or BNB). Equally important is the ability of the BBB to control the influx of pharmaceutical compounds into the CNS parenchyma, thus regulating the efficacy and side effects associated with analgesic and antiinflammatory drugs (Sanchez-Covarrubias et al., 2014).

A clear understanding of the structural and functional changes that occur in the BBB following peripheral injury/chronic pain will provide insights into the molecular mechanisms and pathophysiological profile of different clinical pain disorders, which would permit the development of more effective and perhaps safer therapeutic approaches for chronic pain management.

## HOW DOES INFLAMMATORY PAIN DISRUPT THE BBB?

There is accumulating evidence that inflammatory pain states produce significant changes in the BBB permeability (**Table 1**). This may affect the delivery of therapeutic components to the brain, with great impact in the dosing regimens commonly used to treat patients with chronic pain disorders. In one of the pioneer studies investigating the effects of peripheral inflammation in the BBB function *in vivo*, three different models of subcutaneous inflammatory pain were examined (Huber et al., 2001). The results showed significantly higher distribution of sucrose, a membrane-impermeant marker, into the cerebral hemispheres after peripheral inflammation produced by subcutaneous injections of formalin, λ-carrageenan, or CFA, representing acute, short-term and long-term models of inflammatory pain, respectively. Moreover, peripheral inflammation altered the expression of TJ proteins. ZO-1 expression was significantly amplified in all models analyzed, while occludin was significantly diminished in the groups treated with λ-carrageenan or CFA. The decrease of occludin expression reported in that study was later confirmed in a model of chronic inflammatory pain, using CFA as the inducer agent (Brooks et al., 2005). The same study reported a huge increase in the expression of claudin-3 (450%) and claudin-5 (615%). Nevertheless, changes in ZO-1 could not de demonstrated. The discrepancies between both studies might be explained by differences in the methodologies adopted. In another work, using the CIP model, paracellular permeability to [14C] sucrose was detected in the BBB, which was also paralleled by altered expression of occludin and ZO-1. However, intravenous administration of λ-carrageenan did not significantly impact the BBB permeability, indicating that the change in [14C] sucrose permeability was due to either CIP induced inflammatory or neuronal modulation of TJ (Huber et al., 2002). Furthermore, specific regional microglia activation, measured by OX42 immunoreactivity, and changes in ICAM-1 expression have been shown after CIP (Huber et al., 2006). Increased ICAM-1 expression, associated with microglia activation, has been demonstrated in central-mediated cerebral inflammation (Kyrkanides et al., 2002) and several studies have highlighted the importance of microglia to the mechanisms of neuropathic (Raghavendra et al., 2003; Tsuda et al., 2003; Coull et al., 2005; Ji and Suter, 2007; Saegusa and Tanabe, 2014) and acute inflammatory pain (Svensson et al., 2003; Ji et al., 2013). Nonetheless, it is important to mention that there is evidence that the augmented reactivity of astrocytes or microglia alone, without simultaneous changes in the BSCB or BBB, is not sufficient to generate and maintain a chronic pain state, after direct lesion, or nervous system disease. This was demonstrated in an experimental model of perispinal inflammation induced by the TLR-2 agonist zymosan (Tenorio et al., 2013). Remarkably, the previously reported regional effects of CIP in ICAM-1 expression and microglia activation occurred in brain areas that have been extensively reported to be involved in pain processing and modulation, such as the thalamus, frontal, and parietal cortices (Apkarian et al., 2004; DaSilva et al., 2007a,b, 2008, 2012; DosSantos and DaSilva, 2011; DaSilva and DosSantos, 2012; DosSantos et al., 2012a; Wager et al., 2013), leading to the hypothesis that the alterations seen in the BBB after CIP are possibly driven by a central-mediated response conducted through the spinothalamic tract. This hypothesis was further confirmed in a study showing that CIP-induced changes in the BBB integrity can be prevented by a perineural injection of bupivacaine 0.75%, implying that nociceptive input is necessary to enhance the BBB permeability in λ-carrageenan-driven inflammatory pain (Campos et al., 2008). Interestingly, the same study showed that bupivacaine nerve block also decreased the thermal allodynia and prevented variations in the expression of TJ proteins occludin, ZO-1, and claudin 5, but did not alter the paw edema formation following λ-carrageenan injection. In summary, the results indicate that the blockade of nociceptive input inhibits the functional perturbations in the BBB barrier under inflammatory pain conditions.

**Table 1:** Main findings of studies investigating changes in the BBB/BSCB associated with inflammatory pain.

Barrier	Model	Main outcomes	Reference
BBB	Inflammatory pain, produced by subcutaneous injection of CFA, λ-carrageenan (CIP) or formalin, in sprague–dawley rats.	Peripheral inflammation led to an increase in the uptake of sucrose into the cerebral hemispheres, in all models studied. Western blot revealed changes in the TJ protein expression during peripheral inflammation. Occludin decreased in the groups treated with λ-carrageenan or CFA, while ZO-1 expression was increased in all inflammatory pain models. On the other hand, Claudin-1 protein expression did not change throughout the experiment.	Huber et al. (2001)
BBB	Chronic inflammatory pain, using CFA, in sprague–dawley rats.	Decrease in the expression of Occludin. Significant increase in the expression of claudin-3 (450%) and claudin-5 (615%) were also demonstrated, but the same results were not obtained with zonula occluden-1.	Brooks et al. (2005)
BBB	CIP, in sprague–dawley rats.	Increase in ICAM-1 RNA and protein expression in the thalamus, frontal, and parietal cortices; which were correlated with augmented expression of activated microglia.	Huber et al. (2006)
BBB	CIP and perineural injection of bupivacaine, in sprague–dawley rats.	Changes in the BBB integrity induced by CIP were prevented by a perineural injection of bupivacaine. This data suggests that nociceptive input is necessary to the increased BBB permeability found in λ–carrageenan models of inflammatory pain.	Campos et al. (2008)
BBB	CIP and capsaicin, in sprague–dawley rats.	Significant changes in occludin protein were observed in the lumbar spine after λ-carrageenan but not after capsaicin administration. Simultaneously, significant amounts of immunoglobulin G were seen in the lumbar and thoracic segments of the spinal cord	Xanthos et al. (2012)
BBB	CIP, in sprague–dawley rats.	Structural changes in P-gp.	McCaffrey et al. (2012)
BSCB	Perispinal inflammation induced by zymosan, in mice.	Perispinal inflammation led to changes in the reactivity of resident astrocytes and microglia within the spinal cord but maintained the integrity of the BSCB. Chronic pain did not develop.	Tenorio et al. (2013)
BBB	CIP and diclofenac treatment, in sprague-dawley rats.	Increased P-gp expression following peripheral inflammatory pain and also after diclofenac treatment. Both peripheral inflammatory pain and diclofenac treatment alone increased P-gp eﬄux activity, leading to a reduced morphine brain uptake. Analgesia produced by morphine was significantly reduced in animals pretreated with diclofenac, when compared to those that received diclofenac and morphine concurrently.	Sanchez-Covarrubias et al. (2014)

According to a recent study, peripheral inflammatory hyperalgesia is also responsible for a dynamic redistribution of P-gp and caveolin-1 between endothelial subcellular compartments at the BBB (McCaffrey et al., 2012). P-gp is described as the major eﬄux transporter at the BBB. It combines ATP hydrolysis and drug eﬄux to extrude drugs against concentration gradients (Miller, 2010). In addition, it has been stated that increased functional activity of P-gp during inflammatory hyperalgesia leads to a greater eﬄux transport of morphine, which could explain the reduced ability of this drug to gain access to the brain under inflammatory pain conditions (Seelbach et al., 2007). Hence, changes observed in the P-gp function after λ-carrageenan injection, have a potential therapeutic implication, regarding the delivery of analgesic drugs to the CNS, in particular opioids peptides, as well as other classes of pharmacological agents applied to treat peripheral inflammatory pain disorders. In addition to P-gp, it seems that MRP4, another type of ABC transporter and target of some NSADs, is also important for inflammatory pain (Lin et al., 2008).

Despite the mounting evidence linking BBB disruption and inflammatory pain, it is still controversial whether similar events take place at the level of BSCB. For example, in one paper the extravasation of Evans Blue, a dye that is classically used to measure the BBB/BSCB integrity, was reported after 48 h of carrageenan-induced inflammation (Gillardon et al., 1997) while in other studies, carrageenan- or CFA-induced inflammation apparently did not elicit Evans Blue dye leakage (Lu et al., 2009; Echeverry et al., 2011). On the other hand, it seems that morphine penetration in the spinal cord is facilitated by CFA or carrageenan administration (Lu et al., 2009). In one experiment, testing the effects of carrageenan (which produced mechanical and heat hyperalgesia that peaked at 3–24 h and lasted for 72 h) or capsaicin (which induced mechanical hyperalgesia, with peak at 2–3 h and lasting for 24 h) on the BSCB, significant alterations in endothelial cell occludin protein were seen in the lumbar spine, with a delayed onset of 72 h after intraplantar carrageenan administration. However, the same alteration was not repeated after intraplantar administration of capsaicin, which was intended to produce neurogenic inflammation. Subcutaneous injection of carrageenan did not generate significant effects on occludin protein either, illustrating that the changes observed were due to peripheral inflammation rather than a systemic inflammatory effect (Xanthos et al., 2012). The same study also tested the effects of intraplantar carrageenan using IgG extravasation in the spinal cord, another method to analyze BSCB breakdown. At the same time point that changes occurred with occludin, significant quantities of immunoglobulin G were found in the lumbar and thoracic segments of the spinal cord, probably owing to extravasation. Nonetheless, acute administration of Evans Blue dye or sodium fluorescein was not detected in the CNS parenchyma. Taken together, these findings suggest that peripheral inflammation determines transient changes in BSCB. At first glance, it would not be necessarily linked to the nociceptive signaling. However, the results also highlight the importance of using different methods to assess each particular mechanism responsible for BSCB changes after transient pathologies, and it is possible to speculate that changes induced by capsaicin in this specific study could not be detected by the methodology adopted. It is also important to emphasize that there are significant structural (Ge and Pachter, 2006) and functional (Prockop et al., 1995; Pan et al., 1997) differences between the BSCB and the BBB, including the presence of glycogen deposits in the superficial vessels of the spinal cord, higher permeability to cytokines, and tracers, and the expression of TJ proteins (Daniel et al., 1981; Prockop et al., 1995; Pan et al., 1997; Sharma, 2005; Ge and Pachter, 2006; Radu et al., 2013). All should be considered when evaluating the roles of BBB and BSCB in chronic pain. Therefore, for a more complete evaluation of all CNS barriers under inflammatory pain conditions, it would be highly recommended to compare the behavior of both the BBB and BSCB simultaneously, applying multiple procedures to detect disruption or changes in the permeability of both barriers. Another important fact that must be considered is that the presence of Evans Blue in the brain or spinal cord parenchyma usually occurs with a considerable disruption of the BBB/BSCB. It is likely that the BBB or BSCB permeabilization mediated by inflammatory pain is a transient event, rather than an irreversible phenomenon of disruption or “breakdown” (Brooks et al., 2005; Radu et al., 2013).

There is growing evidence that acute and perhaps chronic inflammatory pain influence the functional and molecular properties of the BBB and BSCB, though probably by distinct mechanisms. The majority of the literature currently available indicates a correlation between increased BBB permeability and altered expression of some transmembrane TJ proteins that collaborate to preserve the BBB integrity. Therefore, it seems that peripheral acute or chronic inflammatory pain leads to a reorganization of the TJ proteins and altered paracellular diffusion, which may alter the delivery of therapeutic analgesic and antiinflammatory substances to the CNS. As such, the increased paracellular permeability and consequent CNS toxicity should be taken into consideration when deciding the dosing regimens for patients affected by chronic inflammatory pain.

It is important to mention that the results of the aforementioned studies linking BBB alterations and inflammatory pain must be interpreted cautiously, since they provide indirect evidence (e.g., changes in TJ protein expression or P-gp function) obtained from experimental models of inflammatory pain, generated artificially, and usually performed during relatively short periods. Hence, translational research is necessary to determine the real impact of BBB dysfunction in chronic diseases with pain of inflammatory origin, including chronic joint inflammation diseases, irritable bowel syndrome, and multiple sclerosis. For example, it has been widely recognized that a BBB pathology is present in multiple sclerosis (Zlokovic, 2008), a concept that is supported not only by experimental (Morrissey et al., 1996; Morgan et al., 2007; Kooij et al., 2009; Reijerkerk et al., 2012) but also clinical data (Plumb et al., 2002; Kirk et al., 2003; Minagar and Alexander, 2003; Leech et al., 2007; Padden et al., 2007; Cramer et al., 2014). BBB disruption has also been reported in CIA, an animal model of RA, implying that this condition could possibly be related to a dysfunctional BBB (Nishioku et al., 2010). As a matter of fact, it seems that RA increases the mortality and morbidity due to cerebrovascular diseases (Watson et al., 2003). Furthermore, it has been reported that the BBB impairment seen in CIA is potentially mediated by S100A4 (Nishioku et al., 2011). This small acidic calcium-binding protein, member of S100 family, is also upregulated in the synovial fluid and plasma of RA patients (Klingelhöfer et al., 2007), which permits a clear connection between the results obtained with the animal model of RA (CIA) and the clinical alterations seen in RA patients. In addition, despite the limited information, the decrease in the expression of TREK1, a TWIK-related potassium channel-1 that is related to pain perception (Alloui et al., 2006) and BBB regulation (Bittner et al., 2013, 2014), after colon inflammation (La and Gebhart, 2011) suggests that the involvement of the central nervous barriers in irritable bowel syndrome should be further explored. In the future, correlations between experimental outcomes and the results of controlled clinical studies will allow researchers to scrutinize the chain of events that take place in the CNS barriers in the presence of chronic inflammatory pain.

## DO THE CNS BARRIERS PLAY A PIVOTAL ROLE IN THE PERIPHERAL AND CENTRAL MECHANISMS OF NEUROPATHIC PAIN?

Neuropathic pain, according to the IASP taxonomy (Merskey et al., 1994), revised in 2012 (http://www.iasp-pain.org/Education/Content.aspx?ItemNumber=1698#Neuropathicpain), is defined as “pain caused by a lesion or disease of the somatosensory nervous system.” It affects approximately 2–3% of the general population (Hall et al., 2006; Bouhassira et al., 2008) with elevated costs to health systems and governments worldwide (Turk, 2002). However, this number can be even higher. Recently the prevalence of pain with neuropathic characteristics has been estimated to be between 6.9 and 10% (van Hecke et al., 2014). Neuropathic pain is considered a clinical description and not a diagnosis. It comprises several disorders, such as radiculopathies, diabetic neuropathies, trigeminal, and postherpetic neuralgia. Although the cellular and molecular mechanisms involved in neuropathic pains have not yet been totally elucidated, there is sufficient evidence that both peripheral and central mechanisms are important. Among them are the release of inflammatory mediators by activated nociceptors at the site of peripheral injury, as well as central sensitization, which encompasses several phenomena, e.g., alteration in glutamatergic neurotransmission/NMDA receptor-mediated hypersensitivity, disinhibition, and neuron-glial interactions (DaSilva et al., 2008; Basbaum et al., 2009; Gustin et al., 2011; DaSilva and DosSantos, 2012; DosSantos et al., 2012b; McMahon, 2013; Wilcox et al., 2013). There is also evidence that vascular events contribute to this process.

Notwithstanding many classical works have focused on the presence of local vascular disturbances following peripheral nerve injury in different experimental models (Myers et al., 1981, 1985; Powell et al., 1991), few studies have explored the specific cellular and molecular processes underlying the vascular events that occur in the presence of neuropathic pain (**Table 2**). To characterize the impact of vascular disturbances in the mechanisms involved in the generation of pain following neuronal damage, a recent study explored the consequences of peripheral nerve injury, produced by a partial ligation of the sciatic nerve, in the BNB functioning (Lim et al., 2014). Overall, the outcomes give rise to the hypothesis that neuropathic pain is, at least in part, associated with higher distribution of molecules that cross a defective BNB, and act at the peripheral nerve already damaged. According to the findings of that study, nerve injury triggers a “breakdown” of the BNB, which is associated with a long-lasting pain behavior. Additionally, It seems that RM play a crucial role in this process. Shortly after peripheral nerve injury, RM cells that are sparsely distributed along the nerves under basal conditions proliferate and start to express the VEGF, which in turn, initiates the “breakdown” of the BNB. This BNB breakdown permits the influx of blood borne macrophages to the endoneurial space. Those infiltrated macrophages produce several cytokines (e.g., IL-1β, TNF-α, and fibrinogen). Fibrinogen probabaly has its effects linked to the activation of TLRs, especially TLR-4. Another interesting finding with possible clinical implications is that ProTX-II, a peptide that blocks NAV1.7 ion channel but does not pass the intact BNB, reversed the mechanical allodynia in the experimental model of neuropathic pain, an effect that is likely restricted to the site of nerve injury. Therefore, substances such ProTx-II with action restricted to peripheral nerves with compromised BNB, and that do not present a significant distribution to uninjured nerves (with preserved BNB), the brain or the spinal cord, emerge as promising therapeutic options in peripheral neuropathies, due to the limited side effects (Lim et al., 2014). Noteworthy, in the specific case of ProTX-II, significant effects would only have been reached in injured nerves, displaying altered NAV1.7 expression. In fact, changes in the NAV1.7 expression have been previously demonstrated in trigeminal neuralgia patients, indicating that such condition could be, at least in part, considered a channelopathy (Siqueira et al., 2009). Mutations in the gene encoding Na_v_1.7 have also been linked to paroxysmal pain disorders (Fertleman et al., 2006; Han et al., 2006), illustrating its importance to the mechanisms of neuropathic pain.

**Table 2:** Summary of recent studies exploring the participation of nervous system barriers (BBB and BSCB) in the mechanisms of neuropathic pain.

Barrier	Model	Main outcomes	Reference
BSCB	Peripheral nerve injury, lidocaine administration and electrical stimulation of the sciatic nerve, in sprague–dawley rats	Peripheral nerve injury produced a transient increase in BSCB permeability. Such event did not occur when lidocaine was administrated at the site of the injury. Increases in the BSCB permeability also occurred after electrical stimulation of the sciatic nerve at intensity sufficient to activate C-fibers but not A-fibers and after application of capsaicin to the nerve. It suggests that the increase of BSCB permeability is driven by activation of TRPV1-expressing primary sensory neurons.	Beggs et al. (2010)
BNB	Neuropathic pain, produced by partial ligation of the sciatic, in mice.	Neuropathic pain related to trauma caused a significant disruption of the BNB. VEGF was expressed by RM. Intraneural injection of serum obtained from animals with nerve injury or treated with LPS generated mechanical allodynia in naive animals. Intraneural injection of fibrinogen also produced a decrease in mechanical thresholds when applied to naive nerves. Such results evidence that blood–borne molecules may contribute to neuropathic pain mechanisms.	Lim et al. (2014)

A disruption of BSCB integrity, illustrated by augmented permeability along with astrocyte activation in the spinal cord, has been shown in an animal model of neuropathic pain, with chronic nerve constriction (Gordh and Sharma, 2006). In a more detailed investigation, Beggs et al. (2010) have shown that both chronic constriction injury (CCI; a model of peripheral nerve injury) and stimulation of healthy primary afferent C-fibers are capable of eliciting a surge in both the BBB and the BSCB permeability, when assessed by Evans Blue dye or HRP. Nevertheless, the most important outcome of that study was that capsaicin applied to an uninjured sciatic nerve mimicked the effects of CCI or C-fibers stimulation, supporting the concept that TRPV1-expressing C-fibers could be responsible for the upsurge in the BSCB/BBB permeability. Further clinical studies would be important to confirm if a similar process occurs in patients aﬄicted by neuropathic pain conditions, such as peripheral neuropathies, trigeminal, and postherpetic neuralgias. If that is the case, it could dramatically affect the penetration of analgesic agents into the CNS, determining the efficacy of those drugs and also central-mediated side effects. In the future, those findings could compose the basis to the development of therapies that purposely augment the BBB permeability by targeting the mediators involved in the afferent-induced opening of the BBB/BSCB. In addition, based on those results, it is possible to speculate that the clinical effects of novel non-pharmacological treatments that have been applied to treat neuropathic and other pain conditions, such as tDCS (Fregni et al., 2006; Antal and Paulus, 2011; DosSantos et al., 2012a) or TMS (Marlow et al., 2013; Leung et al., 2014) could be associated with transient changes in the nervous system barriers. There is recent evidence that endogenous opioids modulate the analgesia produced by those methods of non-invasive brain stimulation, through direct or indirect activation of brain areas important for opioid-mediated anti-nociception, such as the PAG (de Andrade et al., 2011; DosSantos et al., 2012a, 2014; Taylor et al., 2012). Future therapeutic protocols, combining non-pharmacological and pharmacological agents could optimize the analgesic effects obtained with single therapies. Indeed, TENS, which reduces secondary hyperalgesia by activation of opioid receptors (Sluka et al., 1999; Kalra et al., 2001), has been successfully combined with clonidine, an a2-adrenergic agonist, to provide effective reduction of hyperalgesia in an animal model of peripheral inflammation (Sluka and Chandran, 2002). Moreover, in a pilot clinical study, prolonged pain relief was achieved by combining tDCS with a NMDA agonist (D-cycloserine) in a case of orofacial pain refractory to pharmacological treatment (Antal and Paulus, 2011), which could perhaps be linked to transient changes in the BBB or BSCB.

## IS MIGRAINE PATHOPHYSIOLOGY CORRELATED TO A BBB DYSFUNCTION?

It has been estimated that approximately 11–12% of adults suffer from migraine headaches (Rasmussen, 1995; Stovner et al., 2007). The majority of patients report moderate to severe pain during the attacks, with great impact in the quality of life (Lipton et al., 2007). Migraine presents two subtypes, migraine with aura and migraine without aura (Silberstein et al., 2005). Although a large number of recent studies have tried to establish the migraine pathophysiology (Bhaskar et al., 2013; Noseda and Burstein, 2013; Sarrouilhe et al., 2014; Thissen et al., 2014), the role of the neural and vascular mechanisms in this process has been largely discussed in the literature, (Asghar et al., 2011; Grände et al., 2014). As a matter of fact, there is still a debate whether the source of the pain is in the nerves around the cranial arteries, CNS or both (Goadsby et al., 2009; Olesen et al., 2009).

It has been generally accepted that CGRP plays an important role in the migraine pathophysiology (Bell, 2014). CGRP is expressed throughout the CNS, particularly the striatum, amygdala, colliculi, and cerebellum, as well as the peripheral nervous system (Edvinsson, 2008). Recently, CGRP receptor antagonists have emerged as promising drugs to treat migraine. They could act either by blocking CGRP-induced vasodilation of meningeal blood vessels or inhibiting CGRP-mediated pain transmission in the CNS (Bell, 2014). Other approaches to block CGRP effects include the use of CGRP antibodies (Zeller et al., 2008), or specific CGRP-binding RNA-Spiegelmer (Denekas et al., 2006). The fact that CGRP receptor antagonists, such as olcegepant and telcagepant, apparently require very high doses to produce significant clinical effects in migraine patients, raises the possibility that those promising components have to cross the BBB in order to exert their effects (Tfelt-Hansen and Olesen, 2011; Bell, 2014). Thus, according to some authors, it could support the concept that CNS mechanisms are predominantly involved in the migraine pathophysiology (Tfelt-Hansen and Olesen, 2011). In fact, DaSilva et al. (2003, 2007b) have previously demonstrated specific cortical neuroplastic changes in migraine patients. Conversely, the results of a functional neuroimaging study have indicated that changes in cortical blood flow, measured by BOLD signal variations, occur during episodes of migraine with aura. In addition, dilatation of both extracranial (middle meningeal) and intracranial (middle cerebral), as demonstrated by high-resolution direct MRA, has been shown after a migraine attack induced by infusion of CGRP. Remarkably, headache and vasodilatation occurred at the same side and the administration of sumatriptan, a selective antimigraine drug, not only reduced the pain but also resulted in contraction of the middle meningeal artery (Asghar et al., 2011). Collectively, those results suggest a key role of cranial blood vessels in the migraine pathophysiology. In fact, meningeal arteries lack BBB and represent much more permeable structures, compared with cortical vessels (Edvinsson and Tfelt-Hansen, 2008; Grände et al., 2014). There have been considerable advances in the understanding of the sequence of events that lead to a migraine headache. Nevertheless, the specific structural and functional alterations that occur in the brains of patients affected by this disorder still need clarification and BBB dysfunction has emerged as a possible mechanism.

Although mild BBB opening has been previously reported in a patient suffering a severe attack of familial hemiplegic migraine type II (Dreier et al., 2005), the occurrence of BBB opening or disruption during a migraine headache is still a matter of debate (Radu et al., 2013). Migraine, as well as other neurological disorders (which are out of the scope of this study) such as epilepsy and cerebrovascular diseases, are characterized by a phenomenon known as CSD (Martins-Ferreira et al., 2000). CSD is a self-propagating wave of neuronal and glial depolarization first described by Leão (1944) in the mid-forties . Brain edema and plasma protein leakage, concomitant with altered expression of proteins that are important to maintain the BBB integrity, such as the EBA, ZO-1, and laminin (substrate protein of metalloproteinases – MMPs), were demonstrated in an animal model of CSD (Gursoy-Ozdemir et al., 2004). In addition, albumin leakage was suppressed by the injection of the matrix metalloproteinase inhibitor GM6001, but did not occur in MMP-9-null mice. It clearly indicates that the BBB disruption associated with CSD depends on the MMP-9 activity. Although those results cannot be considered exclusive of migraine, but rather related to the CSD phenomenon (that also participates in the migraine mechanisms), elevated plasma levels of MMP-9 have been reported in migraine patients (Leira et al., 2007; Imamura et al., 2008; Martins-Oliveira et al., 2012) and MMPs, especially MMP-2 and MMP-9, have been linked to BBB disruption, as well as augmented influx of inflammatory cells into the CNS (Rosenberg et al., 2001; Gurney et al., 2006; Yang et al., 2007; Bernecker et al., 2011). Furthermore, it has been suggested that MMMP-2 plasma concentrations are higher in migraine with aura than in migraine without aura (Gonçalves et al., 2013) and increased MMP-9 activity has been reported in women with migraine without aura (Martins-Oliveira et al., 2012), suggesting that distinct mechanisms are involved in each form of migraine. Nonetheless, the participation of MMP-9 in the migraine pathophysiology is not completely accepted. According to one study, plasma levels of MMP-9 should not be used as a biomarker of migraine with aura (Ashina et al., 2010). In contrast, the reduction in the plasma concentrations of MMP-3 found during the early phase of headache migraine attacks suggest that this isoform should be further investigated in migraine sufferers. However, the most important information derived from those works is that MMPs might actively contribute to the migraine pathophysiology, and perhaps other types of primary headaches, in a mechanism involving CSD and BBB disruption. Nonetheless, it is important to state that not all primary headaches and subtypes of migraine are necessarily related to CSD, MMPs, and BBB dysfunction and that many other mechanisms can play a role in each particular condition. For example, there are studies showing that gap junctions take part in the migraine pathophysiology, being promising targets for future treatments (Sarrouilhe et al., 2014).

Finally, an ischemic and reversible “breakdown” of the BNB caused by a vasospasm of the vasa nervorum at the brainstem REZ of the oculomotor (III), trochlear (IV), or abducens (VI) nerve has been recently proposed as the pathogenic theory to explain the clinical and neuroimaging findings of OM (Ambrosetto et al., 2014). This is a rare form of episodic migraine-like headache attacks, accompanied, or followed by ophthalmoplegia related to paresis of the one or more of the following cranial nerves: III, IV, or VI. This theory seems to provide a reasonable explanation to the reversible focal thickening and enhancement of the cisternal tract at the REZ of the cranial nerve involved, usually the III, especially when occurring in children (Miglio et al., 2010; Gelfand et al., 2012). An intriguing fact is that the same alterations are not observed in the adult form of OM (Lal et al., 2009). According to this theory, the discrepancies between children and adults regarding the MRI findings in OM could reflect a differential maturation, and consequently the effectiveness of the BBB in children and adults (Ambrosetto et al., 2014).

Overall, the current literature points toward an increase in the permeability or perhaps a “breakdown” of the BBB, with vascular leakage in migraine patients, during the headache attack. This process could be triggered by CSD, in an MMP-dependent pathway (**Table 3**). Defining the pathophysiologic mechanisms that trigger a migraine attack, especially regarding the changes that occur in the BBB permeability are crucial not only to characterize the cascade of events that occur during its ictal phase, but also to provide better treatment choices, with lower side effects, for such a debilitating disorder.

**Table 3:** Direct and indirect evidence that migraine pathophysiology is also correlated to a BBB dysfunction.

Barrier	Model	Main outcomes	Reference
BBB	Cortical spread depression (CSD) model, in sprague–dawley rats and mice.	Direct evidence: brain edema and plasma protein leakage, associated with altered expression ZO-1, EBA, and immunoreactive laminin. Albumin leakage was suppressed by the injection of the matrix metalloproteinase inhibitor GM6001 and was not found in MMP9-null mice. Such results indicate that the BBB disruption related to CSD depends on the MMP-9 activity.	Gursoy-Ozdemir et al. (2004)
BBB	Familial hemiplegic migraine patients.	Direct evidence: quantitative analysis of gadolinium-enhanced MRI showed a mild, but significant, left-hemispheric opening of the BBB, preceding cortical edema.	Dreier et al. (2005)
BBB	Migraine patients	Indirect evidence: no differences in MMP-9 and TIMP-1 levels were found between ictal and interictal periods. However, lower plasma levels of MMP-3 were observed in the external jugular and cubital vein during migraine attacks. Such results suggest that plasma levels of MMP-9 might not be the most recommended biomarker of BBB disruption in migraine without aura. On the other hand, MMP-3 levels should be further investigated.	Ashina et al. (2010)
BBB	Migraine patients	Indirect evidence: higher MMP activity was associated with migraine, independent of aura symptoms.	Bernecker et al. (2011)
BBB	Migraine patients	Indirect evidence: patients presenting migraine without aura showed increased plasma concentrations of MMP-9 concentrations than migraine with aura patients.	Martins-Oliveira et al. (2012)
BBB	Migraine patients	Indirect evidence: patients with migraine with aura exhibited grater plasma concentrations of MMP-2 and MMP-2/TIMP-2 ratios than patients with migraine without aura and controls. CC genotype for C^-735^T polymorphism and the CC haplotype were linked to higher plasma MMP-2 concentrations in the migraine with aura group.	Gonçalves et al. (2013)

## HOW CAN WE MODULATE THE BBB IN ORDER TO IMPROVE THE DELIVERY OF ANALGESIC COMPOUNDS?

The majority of the substances currently available to treat moderate to severe chronic pain (e.g., opioids, anticonvulsants, and antidepressants) have their use limited due to the extensive side effects reported. In addition, tolerance and dependence can be developed over time, mainly with opioid analgesics (e.g., morphine, codeine, oxycodone, and tramadol; McMahon, 2013). Tolerance, for instance, prevents the long-term administration of opioid agonists. Notwithstanding it has been recognized that some complex phenomena, such as mu-opioid receptor desensitization, impaired recovery from desensitization, and impaired recycling after endocytosis (Williams et al., 2013) are associated with morphine tolerance, it is possible that part of the BBB components (e.g., pericytes and astrocytes) also play a role in this process (Chen et al., 2012; Luk et al., 2012). Not surprisingly, amitriptyline, a tricyclic antidepressant largely prescribed for pain control, especially in chronic neuropathic pain disorders, has been shown to attenuate astrocyte activation and consequently morphine tolerance (Huang et al., 2012). Indeed, one the most important concerns in the treatment of inflammatory as well as neuropathic pain is the deleterious drug–drug interaction when other substances (e.g., non-steroidal anti-inflammatory, NSAIDs) are combined with opioids analgesics, resulting in ineffective drug dosing. This is especially important, because chronic pain management often requires the concurrent administration of multiple pharmacological agents (Sanchez-Covarrubias et al., 2014). For instance, NSAIDs are frequently co-administrated with opioids to treat postsurgical pain (Oderda, 2012).

Particularly important in this context, is the P-gp, since it constitutes one of the most important obstacles to the delivery of pharmacological agents to the CNS in several disorders, such as epilepsy, HIV, and Alzheimer’s disease (Ronaldson et al., 2008; Hartz et al., 2010; Potschka, 2012). As previously discussed in this text, a higher expression of P-gp observed in a model of inflammatory pain after λ-carrageenan injection, correlates to a lower transport of morphine in the CNS uptake, which is related to a significant reduction of its analgesic efficacy (Seelbach et al., 2007). Interestingly, not only inflammation but also diclofenac administration has been proved to cause a significant increase of P-gp expression in rat brain microvessels. Additionally, sprague-dawley rats that were pretreated with this drug revealed a lower morphine uptake (Sanchez-Covarrubias et al., 2014). One possible explanation is the drug–drug interaction between NSAIDs and opioids, with a modulatory effect of P-gp. Nevertheless, more data is needed to confirm this hypothesis.

In addition to P-gp, it has been recognized that NSAIDs also interact with other ABC transporters, mainly MRP4, and possibly MRP1 (Reid et al., 2003; Rosenbaum et al., 2005; de Groot et al., 2007). The data available supports that MRP4 has the ability to produce a cellular release of prostaglandins and that some of the most commonly prescribed NSADs (e.g., indomethacin, indoprofen and ketoprofen) act not only by inhibiting the synthesis of prostaglandin, but also by inhibiting its release, acting at the level MPR4 transporter (Reid et al., 2003).

Non-steroidal anti-inflammatory drugs are known to cross BBB. However, according to some studies indomethacin shows a greater passage through the BBB when compared to other NSAIDs (Eriksen et al., 2003; Parepally et al., 2006). As a matter of fact, this drug is the first line therapy in the treatment of some headaches, such as paroxysmal hemicrania and hemicrania continua. The efficacy of indomethacin in those disorders is so high that it is applied as a tool for differential diagnosis of those forms of primary headaches and a positive response to indomethacin is mandatory for a definitive diagnosis of hemicrania continua and paroxysmal hemicrania (Casey and Bushnell, 2000; Summ and Evers, 2013). Indomethacin is also recommended to treat other primary headaches (e.g., stabbing headache and primary cough headache; Merskey et al., 1994; Summ and Evers, 2013). The capacity to interact with MRP4 (Reid et al., 2003) and possibly MRP1 (de Groot et al., 2007), and consequently its high ability to cross the BBB, are probably crucial characteristics that determine the significant clinical efficacy of indomethacin in primary headaches (Summ and Evers, 2013). Although more information is needed regarding the interactions between NSAIDs and the BSCB, it has been reported that in rats submitted to spinal cord injury, a pretreatment with NSAIDs (e.g., indomethacin or ibuprofen) not only attenuates the changes that occur in the spinal cord-evoked potentials immediately after trauma but also contributes to the reduction of edema formation and BSCB permeabilization (Sharma and Winkler, 2002).

Finally, some strategies have been applied to improve the delivery of therapeutic compounds to the CNS. For example, a conjugate of Angiopep-2 and neurotensin, called ANG2002, induced a dose–dependent analgesia, in a formalin model of persistent pain (Demeule et al., 2014). The regulated and reversible opening of the BNB has also been explored in order to develop new strategies to enhance drug delivery to the peripheral nervous system, improving the efficacy and reducing the undesirable central effects of some analgesic drugs, including opioids (Hackel et al., 2012). Though the selective blockade of nociceptive fibers at peripheral sites of injury by analgesic drugs is prevented by the BNB (Radu et al., 2013), it seems that the BNB is already disrupted in cases of peripheral nerve injures (Lim et al., 2014). Thus, the development of compounds with action limited to the peripheral nervous system would be of particular interest.

## CONCLUSION AND PERSPECTIVES

There is mounting evidence that BBB/BSCB/BNB disruptions participate in the complex mechanisms that initiate or maintain inflammatory, neuropathic pain, and migraine. Regarding migraine, this process could be, at least partially, induced by MMPs. BBB and BNB also play a crucial role in the drug–drug interactions, with great impact in the efficacy as well as central-mediated side effects of analgesic agents, especially opioids peptides. Future perspectives include the complete characterization of specific changes in the nervous system barriers in order to establish the molecular mechanisms of each pain disorder. The development of novel drugs to treat neuropathic pain, with effects restricted to the peripheral nervous system would also be desirable. Finally, the contribution of polymorphisms affecting the components of the BBB and the role of epigenetics in the altered permeability of CNS barriers induced by chronic pain should be further explored.

## Conflict of Interest Statement

The authors declare that the research was conducted in the absence of any commercial or financial relationships that could be construed as a potential conflict of interest.

## ABBREVIATIONS

ABC: ATP-binding cassette
ATP: adenosine triphosphate
BBB: blood–brain barrier
BCRP (Bcrp): breast-cancer resistance protein
BNB: blood–nerve barrier
BOLD: blood oxygenation level-dependent
BSCB: blood–spinal cord barrier
CFA: complete freund’s adjuvant
CGRP: calcitonin gene-related peptide
CIA: collagen-induced arthritis
CIP: lambda-carrageenan-induced inflammatory pain
CNS: central nervous system
CSD: cortical spreading depression
EBA: endothelial barrier antigen
HIV: human immunodeficiency virus
HRP: horseradish peroxidase
IASP: international association for the study of pain
ICAM-1: intercellular adhesion molecule 1
IL-1β: interleukin-1 beta
MMPs: matrix metalloproteinases
MRA: magnetic resonance angiography
MRP (Mrp): multidrug resistance protein
NMDA: *N*-methyl-D-aspartate
NSAIDS: non-steroidal anti-inflammatory drugs
OM: ophthalmoplegic migraine
P-gp: P-glycoprotein
PAG: periaqueductal gray
RA: rheumatoid arthritis
REZ: root entry zone
RM: resident macrophages
tDCS: transcranial direct current stimulation
TENS: transcutaneous electrical nerve stimulation
TJ: tight junction
TLR: toll-like receptor
TMS: transracial magnetic stimulation
TNF-α: tumor necrosis factor-alpha
VEGF: vascular endothelial growth factor
ZO: zonula occludens
